# Immune Hyperactivation in Epstein-Barr Virus (EBV)-Driven T-cell Lymphoproliferation: A Pediatric Case of Hemophagocytic Syndrome

**DOI:** 10.7759/cureus.105501

**Published:** 2026-03-19

**Authors:** Nour El Houda Fakhri, Karima Ryouni, El Alaoui Mounia, Noufissa Benajiba

**Affiliations:** 1 Pediatrics, Harouchi Hospital of Casablanca, Casablanca, MAR; 2 Hematology and Oncology, Hôpital Mère-Enfant Abderrahim Harouchi, Casablanca, MAR; 3 Pediatric Hematology, Centre Hospitalier Universitaire Mohammed VI Oujda, Oujda, MAR

**Keywords:** ebv-associated t-cell lymphoproliferation, epstein-barr virus-associated lymphoproliferative disorders, hematopoietic stem cell transplantation, hlh diagnostic criteria and clinical features, immunosuppressive therapy, pediatric secondary hemophagocytic syndrome, refractory hps

## Abstract

Hemophagocytic lymphohistiocytosis (HLH) triggered by Epstein-Barr virus (EBV)-associated T-cell lymphoproliferation represents a rare, aggressive complication in immunocompetent children, often mimicking lymphoma and leading to cytokine storm and multiorgan failure.​ This case report describes a six-year-old girl presenting with fever, cervical mass, hepatosplenomegaly, and cytopenias. Diagnostic evaluation included imaging (CT scans), serology, EBV polymerase chain reaction (PCR) (viral load 545,238 copies/mL), lymph node biopsy showing cytotoxic T-cell proliferation, and serial labs confirming HLH-2004 criteria (fever, splenomegaly, hypertriglyceridemia, cytopenias, hyperferritinemia >500 ng/mL, and hypofibrinogenemia). Treatment involved antibiotics, rituximab, chemotherapy, and HLH-2004 protocol (etoposide, corticosteroids, and cyclosporine).​ Initial antibiotic therapy addressed *Serratia* superinfection, but the disease progressed with worsening cytopenias (hemoglobin (Hb) 7.2 g/dL, platelets 33,000/μL), ferritin 5,010 ng/mL, and multiorgan lesions on follow-up CT. HLH protocol induced transient remission (ferritin 206 ng/mL, normalized counts), but two relapses during tapering (recurrent fever, cytopenias, and rising ferritin) preceded fatal hematemesis in June 2025, after six months since the beginning of illness. This pediatric case underscores EBV's rare T-cell tropism in non-Asian contexts, diagnostic pitfalls from biopsy mimicry, and familial hints of underlying immunodeficiency. Despite multidisciplinary intervention, poor prognosis highlights the need for early HLH recognition and potential hematopoietic stem cell transplantation (HSCT). Rapid immunosuppression controls progression, but relapses remain common.

## Introduction

Hemophagocytic syndrome (HPS) is a critical condition characterized by excessive immune activation, resulting in widespread inflammation and damage to multiple organs. It is broadly classified into primary (familial) HLH, a genetically determined disorder of cytotoxic lymphocyte function typically presenting in infancy or early childhood, and secondary (acquired) HLH, which occurs in the context of strong immune triggers such as infections, malignancies, or autoimmune diseases. While primary HLH is associated with germline defects affecting cytotoxic pathways (e.g., perforin-mediated killing), secondary HLH reflects a dysregulated but non-hereditary immune response, although genetic susceptibility may still contribute in some cases [1,2].

Diagnosis remains challenging due to nonspecific clinical features. The widely used HLH-2004 criteria establish that a diagnosis can be made either by identifying a pathogenic HLH-associated mutation or by fulfilling at least five of eight clinical and laboratory parameters, including fever, splenomegaly, cytopenias affecting ≥2 cell lineages, hypertriglyceridemia and/or hypofibrinogenemia, hemophagocytosis in tissues, low or absent NK-cell activity, hyperferritinemia, and elevated soluble IL-2 receptor (sCD25) [3].

From an epidemiologic perspective, HLH is considered rare but likely underdiagnosed. Infection-associated HLH is most commonly triggered by Epstein-Barr virus (EBV). EBV-driven HLH typically involves infected B cells; however, EBV-associated T-cell or NK-cell lymphoproliferative disease represents a distinct and particularly aggressive entity. Such cases are reported predominantly in East Asian populations, with markedly lower incidence in Europe and North America, making EBV-driven T-cell disease outside Asia an uncommon and clinically noteworthy presentation [1].

EBV infection, while commonly targeting B lymphocytes, can also provoke abnormal proliferation of T and natural killer (NK) cells, leading to severe immune disturbances. In pediatric patients, such proliferations may trigger secondary HPS, presenting significant diagnostic and therapeutic challenges. Effective management hinges on early recognition, immunosuppressive therapies, and in some cases, hematopoietic stem cell transplantation (HSCT) to achieve disease control and improve outcomes.

In children, it may reveal underlying genetic abnormalities (e.g., primary immunodeficiencies). We describe a pediatric case of secondary HPS due to EBV-associated cervical T-cell lymphoproliferation, highlighting the diagnostic and therapeutic challenges.

This article was previously posted as a poster at the 2025 Somipev annual meeting [4].

## Case presentation

A female child aged six years and two months, born to a non-consanguineous marriage, was the youngest of two siblings, with a healthy brother. The pregnancy was well monitored and carried to term, and delivery was vaginal with good neonatal adaptation. Psychomotor development was normal, and vaccination was up to date according to the national schedule. The family history included two miscarriages in the mother at 12 weeks of gestation; the paternal grandmother had three miscarriages and the death of a daughter at the age of four and a son at the age of five (etiology unknown).

At five years and eight months, she presented with a right lateral cervical mass with fistulization, which evolved in the context of fever and general health deterioration.

Clinical examination revealed that the child was pale and febrile at 39.1°C, hemodynamically and neurologically stable. Abdominal examination revealed splenomegaly measuring 5 cm, associated with hepatomegaly without palpable masses. Cutaneous and mucosal examinations revealed no signs of hemorrhage, purpura, or ecchymosis. Examination of other lymph node areas and neurological examination were unremarkable. The remainder of the somatic examination was unremarkable.

The initial lab workup is presented in Table 1.

**Table 1:** Initial biological workup on the first day of hospitalization WBC: white blood cells, CRP: C-reactive protein, ALT: alanine aminotransferase, AST: aspartate aminotransferase, ESR: erythrocyte sedimentation rate

Parameter	Result	Unit of mesure	Reference range
Hemoglobin	12.3	g/dL	11.5–14.5 g/dL
WBC	4180	/µL	5.0–14.5 × 10⁹/L
Neutrophils	3260	/µL	1.5–8.5 × 10⁹/L
Lymphocytes	490	/µL	1.5–7.0 × 10⁹/L
Platelets	285000	/L	150–450 × 10⁹/L
CRP	16.9	mg/L	<5 mg/L
AST	94	UI/L	20–45 U/L
ALT	27	UI/L	10–35 U/L
Ferritin	560	ng/mL	15–100 ng/mL
ESR	10	Mm/hr	0–10 mm/hr

**Figure 1:**
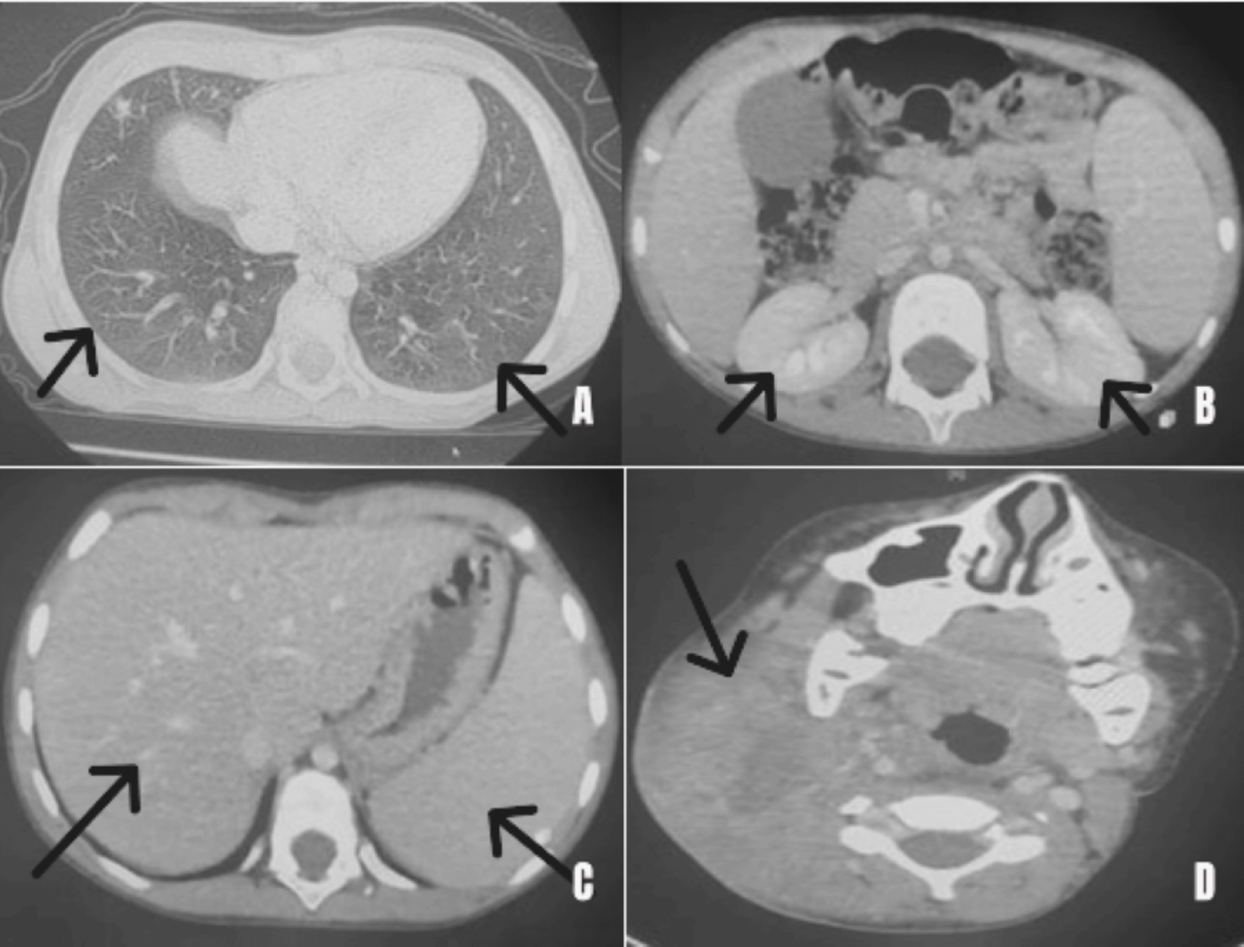
Initial cervico-thoraco-abdomino-pelvic CT scan A: Nodular and micronodular infiltrates with a centrilobular distribution, confluent in some areas, producing a scattered nodular pattern in the bilateral upper lobes and the middle lobe. The largest foci are located in the middle lobe bilaterally (Fowler distribution). B: Enlarged kidneys measuring 9 cm in longitudinal axis on the right and 9.5 cm in longitudinal axis on the left. C: Enlarged liver with a hepatic span of 13 cm, homogeneous in density and with smooth contours, without focal lesions. D: Large mass centered on the right lateral cervical lymph node regions, forming a heterogeneous confluent mass with hypodense areas, associated with streaky infiltration of the submandibular and submental soft tissues.

The patient was initially admitted to the pediatric infectious diseases unit, where a cervico-thoraco-abdomino-pelvic CT scan was performed (09/01/2025). The scan revealed a right cervicofacial lesional process extending to the deep spaces of the ipsilateral face, hepatosplenomegaly, and bilateral nephromegaly (Figure 1).

Swab culture of the serous fluid was positive for *Serratia odorifera*, which was sensitive to ceftriaxone.

The patient was initially treated with triple antibiotic therapy based on ceftriaxone, aminoglycoside, and metronidazole for 10 days, and a lymph node biopsy was performed on 28/01/25, on day 8 of hospitalisation.

**Figure 2:**
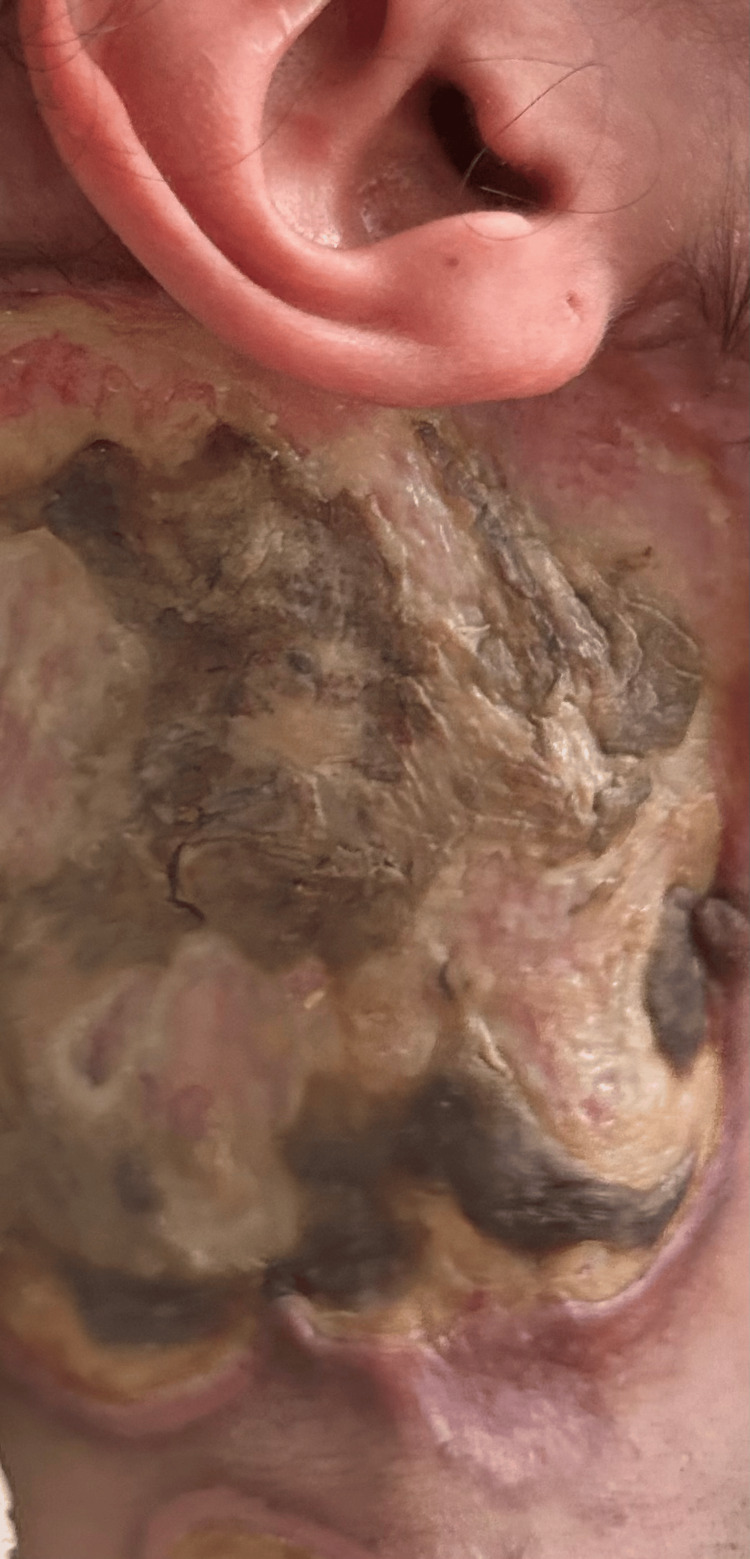
Necrotic lesion at the biopsy site with tissue loss

Three days post-biopsy, the child developed necrosis of the lesion, with tissue loss at the biopsy site (Figure 2).

Tuberculosis workup was negative, and the immunodeficiency workup is shown in Table 2, with HLA-DR of 98%.

**Table 2:** Patient immunodeficiency workup IgG: immunoglobulin G, IgM: immunoglobulin M, IgA: immunoglobulin A, IgE: immunoglobulin E, NK cells: natural killer cells

Parameter	Patient value	Unit	Reference range (age 5–6 years)
Immunoglobulins			
IgG	15.04	g/L	5.0–14.0
IgM	1.17	g/L	0.4–2.0
IgA	2.41	g/L	0.5–2.0
IgE	595.64	IU/mL	< 90
Lymphocyte subpopulations			
CD3+ T cells	422	cells/µL	1,200–2,600
CD4+ T cells	143	cells/µL	650–1,500
CD8+ T cells	245	cells/µL	370–1,100
CD19+ B cells	116	cells/µL	270–860
CD16+ NK cells	205	cells/µL	100–1,000

EBV serology

The results show that the patient is IgG and IgM positive and EBV PCR positive, with a viral load of 545,238 copies/mL. The child was started on day 12 of hospitalisation on immunosuppressive treatment consisting of four rituximab boluses with a decrease in the viral load of EBV to 2,929 copies/mL.

The histopathological result was obtained on day 17 of hospitalization, favoring T-cell lymphoma with a cytotoxic phenotype possibly linked to EBV, although the EBV status could not be evaluated.

Clinically, the patient continued to have prolonged fever resistant to antipyretics and antibiotics, with a worsening general condition. Macrophage activation workup is shown in Table 3.

**Table 3:** Biological workup on day 20 of hospitalization WBC: white blood cells, CRP: C-reactive protein, AST: aspartate aminotransferase, ALT: alanine aminotransferase

Parameter	Result	Unit of measure	Reference range
Hemoglobin	7.2	g/dL	11.5–14.5 g/dL
WBC	977	/µL	5.0–14.5 ×10⁹/L
Neutrophils	267	/µL	1.5–8.5 ×10⁹/L
Lymphocytes	404	/µL	1.5–7.0 ×10⁹/L
Platelets	33000	/L	150–450 ×10⁹/L
CRP	54.3	mg/L	<5 mg/L
AST	320	UI/L	20–45 U/L
ALT	227	UI/L	10–35 U/L
Ferritin	5010	ng/mL	15–100 ng/mL
Fibrinogen	0.6	g/L	2–4 g/L
Triglyceride	3.1	g/dL	<0.7 g/dL

Bone marrow examination results were normal, indicative of a cell-rich smear. Megakaryocytes were present in normal numbers. All cellular lineages were represented, with no blasts or macrophagic cells. Soluble IL-2 receptor (sCD25) and NK-cell activity were not performed due to a lack of resources.

The patient was transferred to our pediatric hematology-oncology unit for suspected reactive EBV-driven cytotoxic proliferation with HLH or peripheral T-cell lymphoma.

A follow-up CT scan (CTAP) on 14/03/25 showed, compared to the exam on 09/01/25, an increased size of the right cervicofacial lesion process, locally advanced consistent with known pathology; increased size and number of pulmonary lesions; appearance of hepatic, pancreatic, and splenic lesions; atypical morphologic lesion in the mid-polar region of the right kidney; and hepatosplenomegaly with bilateral nephromegaly.

She was initially managed as a T-cell lymphoma, with close monitoring of disease progression and treatment response. The patient received chemotherapy consisting of seven days of prednisone (60 mg/m²/day, orally three times a day), vincristine (1 mg/m²/day, once a day and intravenously), cyclophosphamide (300 mg/m²/day, once a day and intravenously), and intrathecal methotrexate and hydrocortisone. Clinical evolution showed deterioration with fever recurrence, increased pallor, rectal bleeding, persistent pancytopenia, hepatic cytolysis, and hyperferritinemia. Given the severity of the condition and the non-response to the chemotherapy, treatment was changed according to the HLH 2004 protocol.

A very good clinical improvement was noted, with fever resolution by day 4 of treatment and improvement in the general condition. Biologically, ferritin decreased to 206 ng/mL on 04/05/2025, fibrinogen to 2 g/L, and normalization of blood counts (Hg 10.6, WBC 9510, and platelets 231,000).

**Figure 3:**
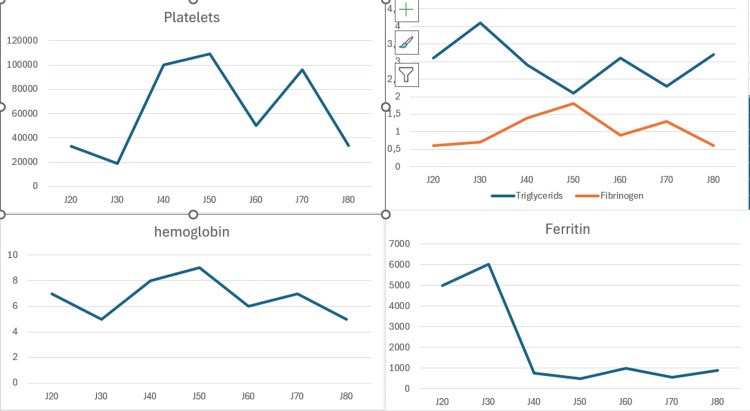
Evolution of the patient’s biological parameters since the beginning of her hospitalization Image generated by the authors with Microsoft Excel (Microsoft Corp., USA)

The course was marked by two reactivation episodes: recurrence of fever, cytopenias, hyperferritinemia, hypofibrinogenemia, and hypertryglyceridemia, as shown in Figure 3, during corticosteroid and etoposide tapering, followed by death in early June after six months of evolution due to massive hematemesis refractory to multiple transfusions of fresh-frozen plasma, platelets, and blood, caused by coagulation disorders from liver failure.

## Discussion

Systemic EBV-positive T/NK-cell lymphoproliferative disorders (LPDs) are rare, aggressive diseases that can affect immunocompetent children. Although EBV classically targets B cells, a subset of cases involves T or NK cells, often with fulminant clinical courses and high mortality [5]. Our patient’s presentation sits squarely in this spectrum and highlights diagnostic and therapeutic challenges in resource-limited settings.

How EBV infects T cells remains incompletely defined. The expression of CD21 (CR2) - the canonical EBV entry receptor on B lymphocytes - has been reported on subsets of immature or rare mature T cells, suggesting a potential entry route; alternative mechanisms likely contribute. Viral persistence within cytotoxic T cells can drive immune hyperactivation, fueling cytokine storms and organ dysfunction [6].

Children usually present with persistent high-grade fever, constitutional decline, and near-constant hepatosplenomegaly, with or without lymphadenopathy. Cytopenias and liver enzyme abnormalities are frequent. Serology is often non-contributory; quantitative EBV PCR is central to confirming viremia and burden. Our case mirrored this phenotype, including a markedly elevated EBV DNA load. The entity aligns with WHO-classified systemic EBV-positive T-cell LPDs of childhood [7]. These diseases, often described in East Asia [8,9], are much rarer in Caucasian children, making our observation particularly significant.

Given the overlap with HLH, early application of the HLH-2004 criteria is essential [10,11]. The absence of demonstrable hemophagocytosis on initial marrow examination does not exclude HLH, particularly early in the disease [12], as was shown in our case presentation.

The family history marked by multiple miscarriages and unexplained early deaths could suggest an unexplored genetic pathology, as suggested by Kimura [13], potentially impairing the T-cell immune response and facilitating viral persistence in T lymphocytes. This was not performed because of resource limitations.

The clinical presentation observed in our patient is consistent with published pediatric cases of systemic EBV-associated T-cell LPD [5,14]. Previously healthy children typically experience a rapidly progressive course with persistent fever, hepatosplenomegaly, cytopenias, and high circulating EBV DNA. Markedly elevated viral load supports active EBV-driven lymphoid proliferation and correlates with severity and poor prognosis in several cohorts. Cervical lymphadenopathy with necrosis, prolonged fever, and hepatosplenomegaly are frequently described. Progressive cytopenias and hepatic involvement underscore the systemic nature of the disease and are commonly attributed to immune-mediated destruction of hematopoietic cells and cytokine-driven marrow suppression during uncontrolled immune activation [6,7].

The development of HLH represents a pivotal, life-threatening complication of EBV-associated T-cell LPDs. EBV is among the most common infectious triggers of secondary HLH in children [5]. Pathophysiology centers on impaired cytotoxic activity of CD8+ T cells and NK cells, leading to macrophage overactivation and excessive cytokine release. Fulfillment of HLH-2004 criteria supports diagnosis even when hemophagocytosis is absent early in the course.

Initial treatment targets rapid immunosuppression and control of hyperinflammation using corticosteroids, etoposide, and calcineurin inhibitors; adjunctive measures such as plasmapheresis can be considered for severe cytokinemic states [15]. Despite protocol-driven therapy, outcomes are often poor, with rapid progression to multiorgan failure, coagulopathy, and hepatic failure. Allogeneic HSCT remains the only potentially curative option for patients who achieve disease control [16], although feasibility is limited by rapid deterioration, as was shown in our case.

## Conclusions

This case illustrates the lethal progression of EBV-associated T-cell lymphoproliferation culminating in secondary hemophagocytic lymphohistiocytosis in an otherwise healthy child. Multidisciplinary vigilance is essential to distinguish lymphoma-like features from true HLH, enabling prompt HLH-2004 initiation despite initial setbacks from superinfection and biopsy complications. While aggressive therapy yields temporary remission, tapering often sparks relapses, with familial history signaling possible hidden immunodeficiencies; advancing cytotoxic testing and stem cell options could enhance outcomes.
